# Postpartum depression: A role for psychedelics?

**DOI:** 10.1177/02698811221093793

**Published:** 2022-05-30

**Authors:** Chaitra Jairaj, James J Rucker

**Affiliations:** 1 Department of Psychological Medicine, Institute of Psychiatry, Psychology & Neuroscience, King’s College London, London, UK; 2 The National Maternity Hospital, Dublin, Ireland; 3 Bethlem Royal Hospital, South London and Maudsley National Health Service Foundation Trust, Beckenham, UK

**Keywords:** Mother–infant relationship, postpartum depression, psilocybin, psychedelics

## Abstract

**Background:**

Postpartum depression (PPD) is a major public health concern and has, at its core, a sense of maternal ‘disconnection’ – from the self, the infant, and the support system. While PPD bears similarities with MDD, there is increasing evidence for its distinct nature, especially with the unique aspect of the mother-infant relationship. Current treatment modalities for PPD, largely based on those used in major depressive disorder (MDD), have low remission rates with emerging evidence for treatment resistance. It is, therefore, necessary to explore alternative avenues of treatment for PPD.

**Objective:**

In this narrative review, we outline the potential therapeutic rationale for serotonergic psychedelics in the treatment of PPD, and highlight safety and pragmatic considerations for the use of psychedelics in the postpartum period.

**Methods:**

We examined the available evidence for the treatment of PPD and the evidence for psychedelics in the treatment of MDD. We explored safety considerations in the use of psychedelics in the postpartum period.

**Results:**

There is increasing evidence for safety, and encouraging signals for efficacy, of psilocybin in the treatment of MDD. Psilocybin has been shown to catalyse a sense of ‘reconnection’ in participants with MDD. This effect in PPD, by fostering a sense of ‘reconnection’ for the mother, may allow for improved mood and maternal sensitivity towards the infant, which can positively impact maternal role gratification and the mother-infant relationship.

**Conclusion:**

Psychedelic assisted therapy in PPD may have a positive effect on the mother-infant dyad and warrants further examination.

## Introduction

The transition to motherhood, termed ‘matrescence’, is a transformative life event involving both positive and negative emotional processes and, with a complex interplay of hormonal, physical, psychological and social factors, is a significant risk factor for the development of postpartum mental illness (Davé et al., 2010; Raphael, 1975). Postpartum depression (PPD), in particular, is recognised as a significant public health concern (Bauer et al., 2014). With emerging evidence for treatment resistance, there is a need for innovative and efficacious treatment for PPD in order to improve wellbeing in the mother–infant dyad (Cepeda et al., 2019). Brexanolone, an analogue of pregnanolone and a positive allosteric modulator of the GABA-A receptor, is the only drug to be developed specifically for the treatment of PPD, and has gained regulatory approval for the treatment of PPD in the United States (Kanes et al., 2017; Meltzer-Brody et al., 2018). Aside from brexanolone, there are no licensed treatment options for PPD, highlighting a need for investigating alternative forms of treatment.

Prohibited from routine clinical use since 1971, research into the use of classic psychedelics, specifically psilocybin and lysergic acid diethylamide (LSD), has resumed since 2000. Early phase clinical trial evidence suggests safety and feasibility of psilocybin given with psychological support in the treatment of depression (Carhart-Harris et al., 2016; Davis et al., 2021), end-of-life distress (Griffiths et al., 2016; Grob et al., 2011; Ross et al., 2016), and substance use disorders (Bogenschutz et al., 2015; Johnson et al., 2014). There is growing evidence for the transdiagnostic applications of psychedelic therapy (Kelly et al., 2021). In this review, we outline the potential therapeutic rationale for serotonergic psychedelics in the treatment of PPD, and highlight safety and pragmatic considerations for the use of psychedelics in the postpartum period.

### Postpartum depression

Perinatal depression is a common illness with prevalence rates ranging from 10% to 20%, with higher incidence across some cultures and in socially disadvantaged populations (Gavin et al., 2005; Jairaj et al., 2019; O’Hara and Swain, 1996; Shorey et al., 2018). Risk factors for developing PPD include a range of psychosocial and biological factors including antenatal depression, childbirth, past history of trauma, intimate partner violence, lack of social support, social deprivation, impaired mother–infant interaction, and adverse life events (Jairaj et al., 2020; O’Hara and McCabe, 2013; Righetti-Veltema et al., 1998; Robertson et al., 2004). In addition to its consequences on maternal mental and physical health, perinatal depression is associated with negative foetal and neonatal outcomes, impaired infant neurodevelopment and attachment, and increased risk of developing depression during adulthood (O’Leary et al., 2019, 2021; Oyetunji and Chandra, 2020; Plant et al., 2015; Slomian et al., 2019). The public health cost of a single case of perinatal depression was reported to be £74,000 in the United Kingdom, with £23,000 of the cost relating to the mother and £51,000 relating to the infant (Bauer et al., 2014). Bearing these protracted human and economic costs in mind, timely and appropriate management of PPD is essential for the well-being of the mother and her infant.

PPD has a heterogeneous presentation with high comorbidity with anxiety (Dennis et al., 2018). Women with PPD can present with pervasive low mood, impaired sleep, appetite, and concentration, feelings of inadequacy or guilt, and a sense of detachment from the infant. Suicidal ideation is common, seen in 20% of women with PPD (Wisner et al., 2013). Irritability and obsessional thoughts, particularly concerning the infant, and thoughts of harm to the infant are also reported in PPD (Spinelli, 2004; Stewart and Vigod, 2019).

The pathophysiology of PPD remains unclear but is thought to be multifactorial with neuroendocrine factors, neurotransmitter changes, inflammation, genetics, and environmental risk factors all playing a role. The abrupt withdrawal of hormones after delivery is thought to precipitate PPD in women with a vulnerability to developing the disorder (Bloch et al., 2000). Dysfunction of the hypothalamic-pituitary-adrenal axis and changes in GABA pathway signalling are implicated in the aetiology of PPD (Licheri et al., 2015; Meltzer-Brody et al., 2011). Low allopregnanolone levels of postpartum are thought to underlie the pathophysiology of PPD in some women, and this hypothesis formed the basis of the development of brexanolone for the treatment of PPD (Meltzer-Brody and Kanes, 2020).

It is debated that PPD is distinct to depression occurring outside the perinatal period, with differing aetiology and therapeutic needs to major depressive disorder (MDD), particularly because of the unique aspect of the mother–infant relationship (Batt et al., 2020; Di Florio and Meltzer-Brody, 2015). While there are clear similarities between PPD and MDD in some areas such as symptomatology, genetic risk factors, decreased activation in reward-related regions, and associations with early life and chronic psychosocial stress, there are also several significant differences (Couto et al., 2015; Elwood et al., 2019; Kimmel et al., 2020; Laurent and Ablow, 2012).

Hormonal factors, change in role to motherhood, and the responsibility of caring for a dependent infant are all specific to PPD. Genetic and hormonal factors are thought to predict the onset of PPD in the early postpartum period (the first 6–8 weeks postpartum), while the unique psychosocial stressors of parenting and infant care predict PPD in the late postpartum period (beyond the first 8 weeks postpartum) (Beck, 2001; Bloch et al., 2003; Figueiredo et al., 2015; Venkatesh et al., 2014). Infant-related symptoms, including feelings of maternal inadequacy and negative perceptions of the infant, are distinct to PPD (Lefkovics et al., 2018). Although research into the neurobiology of PPD is in the early stages, dampened amygdala response and corticolimbic activity, both in early and late PPD, is emerging as a distinct feature (Moses-Kolko et al., 2014; Stickel et al., 2019). While MDD involves a heightened amygdala response to negative stimuli (Fales et al., 2008), PPD is associated with a blunted amygdala response to negative (non-infant related) stimuli (Silverman et al., 2011), which predicts greater self-reported maternal hostility towards the infant (Moses-Kolko et al., 2010). Reduced activation of corticolimbic circuitry involved in emotional salience and threat processing is also a distinct feature of PPD, and is thought to underlie the decreased maternal sensitivity and increased self-reported hostility towards the infant seen in PPD (Moses-Kolko et al., 2014; Pechtel et al., 2013). Although PPD overlaps with MDD in some aspects, it has unique features that may explain why it does not respond adequately to treatment options for MDD in some women.

The therapeutic needs of PPD, while sharing the goals of symptom remission and improved quality of life with MDD, have the additional aim of improving maternal care and the quality of the mother–infant interaction (Batt et al., 2020; Brummelte and Galea, 2016; Di Florio and Meltzer-Brody, 2015). Current treatments for PPD are based on those used in the general adult population, and evidence for antidepressant treatment specific to PPD is limited. Selective serotonin reuptake inhibitors (SSRI) are commonly used, although systematic and Cochrane reviews suggest there is little evidence of efficacy for SSRIs in treating or preventing PPD (Molyneaux et al., 2014, 2018; Sharma and Sommerdyk, 2013). The tricyclic antidepressant nortriptyline was shown to be of similar efficacy to sertraline for treating PPD, although was not effective in preventing PPD (Wisner et al., 2001, 2006). Small studies of bupropion (*n* = 8) and venlafaxine (*n* = 15) in the treatment of PPD have shown positive results but these have not been replicated (Cohen et al., 2001; Nonacs et al., 2005). In one study of treatment outcomes in PPD, only 6.3% of women with PPD received adequate treatment and just 3.2% achieved remission (Cox et al., 2016). There is also evidence for treatment resistance in PPD (Cepeda et al., 2019; Cox et al., 2016). Current MDD treatments are, therefore, not adequately targeting the distinct features of PPD.

Brexanolone, developed specifically for the treatment of PPD, has shown promise in clinical trials. It is administered as an intravenous infusion over a 60-h period. It results in a rapid treatment response with a reduction in depressive symptoms from about 24 h after starting treatment (Meltzer-Brody et al., 2018). Phase 3 trials of brexanolone in PPD have demonstrated a significant Hamilton Depression Rating Scale score reduction of 17.7 to 19.5 points in brexanolone groups compared to 14 points in placebo groups (Meltzer-Brody et al., 2018). Response appears to be maintained for at least 30 days after the infusion. There are no studies evaluating response beyond this period. Adverse effects include excessive sedation and sudden loss of consciousness during drug administration, requiring admission to a medical ward for brexanolone treatment (Meltzer-Brody et al., 2018). The cost of administering brexanolone is reported to be US$34,000, not including hospital admission costs (Faden and Citrome, 2020). The high cost and prolonged period of admission required for brexanolone treatment are barriers to its accessibility and generalisability.

Postpartum maternal mental disorders can impact on infant development and future well-being, in part due to adverse effects on mother–infant interaction and attachment (Reck et al., 2018; Tronick and Reck, 2009). Maternal sensitivity, characterised by the mother’s ability to accurately perceive, interpret and respond to her infant’s signals, can be compromised in PPD, and can result in an impaired mother–infant relationship (Ainsworth et al., 1971). Antidepressants, although having a positive effect on mood in some women, have no effect on the mother–infant interaction, and the influence of PPD on the mother–infant relationship may continue beyond maternal symptom resolution (Batt et al., 2020; Murray and Cooper, 1997; Stein et al., 1991). Parent–infant psychotherapy has been developed to improve health outcomes in the parent–infant dyad. In addition to the management of parental postpartum psychological symptoms, parent–infant psychotherapy aims to establish stable parent–infant relationship patterns and improve parental sensitivity (Barlow et al., 2016; Mattheß et al., 2021). This intervention has been shown to be effective in alleviating depressive symptoms and improving infant attachment security (Barlow et al., 2015; Tsivos et al., 2015). Targeting maternal behaviour in the treatment of PPD is important not only for maternal role gratification and self-efficacy but also for ensuring optimal infant development (Batt et al., 2020; Kingston et al., 2012).

In summary, PPD, even in the late postpartum period, appears to have distinct features to MDD with dampened corticolimbic circuitry and amygdala response (Moses-Kolko et al., 2014; Silverman et al., 2011), in addition to the unique aspect of the mother–infant relationship. Current treatment options for PPD, with the exception of brexanolone, assume similarities with MDD and disregard the differences, leading to poor efficacy and treatment resistance. Persistent PPD can impact negatively on the mother–infant relationship, leading to suboptimal outcomes for the dyad. This highlights a rationale for exploring treatments with novel mechanisms for PPD.

## Overview of psychedelic research

There is a long history of ritualistic use of the tryptamines psilocybin and N,N-dimethyltryptamine, the main psychedelic component of ayahuasca. The first synthetic psychedelic known to the Western world was LSD. There was a period of extensive research with suboptimal methodologies in the 1950s and 1960s into the use of classic psychedelics for the treatment of anxious and depressive disorders, substance use disorders, functional neurological disorders, and distress in terminal cancer, with results that (albeit clinician-judged) were often encouraging (Butler et al., 2020; Dyck, 2006; Grof and Halifax, 1977; Rucker et al., 2016; Weston et al., 2020). However, LSD, in particular, diffused from the clinic into recreational use. Concerns about serious adverse effects arose, and classic psychedelics like LSD and psilocybin were included in international conventions and subsequent national laws that prevented routine medical use (Rucker et al., 2018).

Since the turn of the millennium, clinical research with classic psychedelics (particularly psilocybin) has resumed despite legal restrictions remaining unchanged. Trials were initially conducted in healthy volunteers (Vollenweider et al., 1997) and patient trials commenced later (Moreno et al., 2006). A pooled analysis of eight double-blind placebo-controlled experimental studies conducted between 1999 and 2008 included 110 healthy subjects who received one to four oral doses of psilocybin (45–315 µg/kg body weight) (Studerus et al., 2011). Acute adverse drug events, characterised by strong dysphoria and/or anxiety/panic, occurring in a small proportion of subjects in the two largest dose groups, were managed with interpersonal support. Follow-up of participants suggested that there was no subsequent drug abuse, prolonged psychosis or long-term functional impairment. The largest randomised, double-blind, placebo-controlled study measuring cognitive safety of psilocybin in healthy volunteers completed in 2019 reported no negative impact on a range of cognitive scales, no serious adverse events in participants followed up to 12 weeks after psilocybin dosing, and no adverse events leading to participant withdrawal from the trial (Rucker et al., 2022). Although psilocybin has low abuse potential and is well tolerated, dysphoric experiences (‘bad trips’) can sometimes occur (Johnson et al., 2018; Nutt, 2015) and are minimised in psychedelic trials by excluding participants with a personal or family history of psychotic illness, ensuring the establishment of rapport with the guides or therapists before the dosing session and providing a safe environment for the session (Johnson et al., 2008).

Serotonergic psychedelics exert their action through numerous serotonin receptors (5-HTR), with psilocybin and LSD acting as partial agonists at the 5-HT1R, 5-HT2R, 5-HT6R and 5-HT7R (Nichols, 2004; Rickli et al., 2016). Agonism at the 5-HT2AR is thought to be necessary (but not sufficient) for subjective psychedelic effects. The 5-HT2AR plays a role in cognitive processing, and pre-clinical data suggest that 5-HT2A pathway signalling may be involved in mediating neural plasticity (Carhart-Harris and Nutt, 2017; Ly et al., 2018). However, lisuride, a drug with a high affinity for 5-HT2AR and 5-HT2CR, produces no psychedelic effects (Pieri et al., 1978). This suggests that the functionally selective effects of serotonergic psychedelics on the 5-HT2AR differentially activate downstream second messenger signalling pathways to mediate subjective effects (Flanagan and Nichols, 2018).

Serotonergic psychedelics enhance amygdala activity and reduce default mode network (DMN) and salience network connectivity (Barrett et al., 2020; Carhart-Harris et al., 2017; Lebedev et al., 2015; Roseman et al., 2018). It has been hypothesised that psilocybin increases DMN connectivity in the post-acute phase, followed by a ‘resetting’ and reduction in DMN connectivity, which is proposed to be associated with the therapeutic effects of psychedelics (Carhart-Harris et al., 2017). However, these functional connectivity effects are not specific to classic psychedelics, with 3,4-methylenedioxymethamphetamine and the SSRI sertraline also showing similar results (Klaassens et al., 2015; Müller et al., 2020). This does not exclude the possibility that these effects might be important for the potential therapeutic benefits of psychedelics, although it highlights the constraints of our comprehension of the mechanisms by which psychedelics exert their therapeutic action.

### Psychedelic trials in depression

A systematic review of pre-prohibition studies of classic psychedelics in cases classed as ‘depressives’ or ‘depressive reactions’ reported that clinician-judged improvement occurred in 79.2% of patients (Rucker et al., 2016). Recent trials of psychedelics in MDD have demonstrated safety of psychedelics as a precursor to future randomised controlled trials (RCT), and report encouraging results, albeit with small sample sizes and single centre designs that do not allow generalisation of the results more widely (Table 1).

**Table 1.** Psychedelic trials in depression.

Condition	Study design	Sample size	Psychedelic	Outcomes	Adverse events	Authors, Date
Recurrent depression	Open-label trial	*n* = 6	Ayahuasca	**Day 1:** Statistically significant reduction in HAM-D scores by 62% **Day 7:** 72% reduction **Day 21:** enduring low scores, 45% below baseline	Vomiting	de Osório et al. (2015)
Recurrent depression	Open-label trial	*n* = 17	Ayahuasca	**Day 21:** Significantly lower HAM-D scores, with mean scores of 7.56 (SD = 4.7)	Vomiting, non-significantly increased blood pressure and heart rate	Sanches et al. (2016)
Treatment-resistant depression	Randomised placebo-controlled trial	*n* = 14 (ayahuasca group) *n* = 15 (placebo group)	Ayahuasca Placebo	**Days 1 and 2:** Lower MADRS scores in ayahuasca group compared to placebo **Day 7:** Significantly lower MADRS scores with between-groups effect size of 1.49. Response rate 64% in ayahuasca group	Transient nausea, vomiting and restlessness	Palhano-Fontes et al. (2019)
Treatment-resistant depression	Open-label feasibility trial	*n* = 12	Psilocybin 10 mg and 25 mg, 7 days apart	**1** **week:** Response rate 67% (*n* = 8), and 7 of these patients met criteria for full remission. Depression scores decreased from 21.4 to 7.4, Hedges’ g effect size 3.1 **3** **months:** 58% (*n* = 7) maintained response, 42% (*n* = 5) remained in complete remission. **6** **months:** Depression scores remained low with a 14.9 point mean difference from baseline, effect size 1.2	Transient anxiety, confusion, nausea, headache.	Carhart-Harris et al. (2016, 2018)
Major depressive disorder	Randomised, waiting list-controlled trial	*n* = 27	Psilocybin 20 mg/70 kg and 30 mg/70 kg, 1 week apart. Control arm received same intervention delayed by 8 weeks	**Day 1 after dosing**: rapid reduction in depression scores from 16.7 to 6.3. **1** **week:** depression scores remained low, effect size 2.2. 67% of participants had clinically significant response to intervention. **4** **weeks:** 71% had clinically significant response.	Mild-to-moderate headache and challenging emotions limited to the duration of psilocybin sessions.	Davis et al. (2021)
Major depressive disorder	Double-blind randomised controlled trial comparing psilocybin with escitalopram	*n* = 30 (psilocybin group) *n* = 29 (escitalopram group)	Psilocybin group: Psilocybin 25 mg, 2 doses 3 weeks apart and daily oral placebo for 6 weeks Escitalopram group: Psilocybin 1 mg, 2 doses 3 weeks apart, 6 weeks of oral escitalopram 10–20 mg	**Primary outcome:** Change in QIDS-SR scores – a non-significant between-group difference of two points. **Secondary outcomes:** All other depression rating scales favoured psilocybin but analyses were not corrected for multiple comparisons and no conclusions can be drawn from results.	Similar incidence of adverse events in both groups.	Carhart et al. (2021)

HAM-D: Hamilton Depression Rating Scale; MADRS: Montgomery Asberg Depression Rating Scale; QIDS-SR: Quick Inventory of Depressive Symptomatology (Self-Report).

In an open-label study of a single dose of ayahuasca in six patients with recurrent depression, a 72% reduction in depression scores by day 7 after dosing, with enduring lower scores up to day 21, was reported (de Osório et al., 2015). Another open-label study of 17 patients with recurrent depression dosed with ayahuasca found significantly lower depression scores up to day 21 after dosing (Sanches et al., 2016). Aside from vomiting and non-significantly increased blood pressure and heart rate, participants did not report any adverse events in either open-label trial. More recently, a randomised placebo-controlled trial of a single dose of ayahuasca in 29 participants with treatment-resistant depression was reported (Palhano-Fontes et al., 2019). Participants received either ayahuasca or placebo, a liquid designed to simulate the taste, smell, and consistency of ayahuasca, although without the subjective psychedelic effects. Following the dosing session lasting about 8 h, participants had a psychiatric evaluation, debriefed their experience, and returned home. A rapid antidepressant effect was noted in the ayahuasca group compared to the placebo group, with significantly lower depression scores up to 7 days after dosing and a between-groups effect size of 1.49 (Palhano-Fontes et al., 2019). Aside from transient nausea, vomiting and restlessness, no safety issues were noted in the ayahuasca group. Further research is warranted to demonstrate safety and efficacy of ayahuasca in the treatment of MDD.

An open-label study evaluating the feasibility of psilocybin assisted psychotherapy in 12 patients with moderate to severe treatment-resistant depression was the first modern-day depression trial conducted with psilocybin (Carhart-Harris et al., 2016). Participants, after an initial preparatory session with a therapist, received 10 mg and 25 mg of oral psilocybin 7 days apart. Psychological support was provided during the sessions, with further psychological input at the 1-week follow-up visit. One week after dosing, 67% of participants had achieved complete remission with depression scores dropping from 21.4 to 7.4, and a Hedges’ g effect size of 3.1. At 3-month follow-up, 58% of patients maintained response while 42% remained in complete remission, with an effect size of 2.0. Depression scores remained significantly reduced at the 6-month follow-up, with an effect size of 1.2 (Carhart-Harris et al., 2018). No major safety concerns were reported in this study. As this was an open-label pilot study, efficacy results are likely to be biased and should be treated with caution. However, this study demonstrated that the intervention was possible to deliver in a clinical trial setting, and laid a foundation for more rigorous forms of trial design.

A randomised, waiting list-controlled trial of psilocybin-assisted therapy in 27 participants with MDD aimed to investigate the effect of psilocybin therapy in MDD (Davis et al., 2021). Participants in one arm of this study received a moderate dose of psilocybin (20 mg/70 kg) followed by a high dose (30 mg/70 kg) 1 week later. The control arm received the same intervention delayed by 8 weeks, in order to differentiate effects of psilocybin intervention from spontaneous symptom improvement. Both groups received supportive psychotherapy. Depression scores showed rapid reduction on day 1 after the first psilocybin dosing, from 16.7 to 6.3. Following the second psilocybin dose 1 week later, depression scores remained low at 4 weeks, with a Cohen *d* effect size of 2.2. In the overall sample, 67% of participants had clinically significant response (defined as 50% or greater reduction in depression scores from baseline) to the intervention at week 1, increasing to 71% at week 4. These results suggest that psilocybin may produce a rapid antidepressant response with sustained remission up to 4 weeks after dosing. This was a single centre trial with carefully selected participants, and replication of these results in multi-centre RCTs is needed.

A phase 2, double-blind RCT comparing psilocybin with the SSRI escitalopram in a cohort of 59 participants with moderate-to-severe MDD was recently reported (Carhart-Harris et al., 2021). Patients assigned to the psilocybin group (*n* = 30) received two separate doses of psilocybin 25 mg 3 weeks apart, in addition to 6 weeks of daily oral placebo. Patients randomised to the escitalopram group (*n* = 29) received two separate doses of psilocybin 1 mg 3 weeks apart, in addition to 6 weeks of oral escitalopram (10–20 mg). Both groups received psychological support. The primary outcome was a change from baseline score on the Quick Inventory of Depressive Symptomatology-Self-Report (QIDS-SR-16). The incidence of adverse events was similar among the two groups. The mean change in QIDS-SR-16 scores from baseline to week 6 were −8.0 ± 1.0 in the psilocybin group and −6.0 ± 1.0 in the escitalopram group, with a between-group difference of two points (95% CI: −5.0 to 0.9). In comparison to previous trials with relatively large effect sizes for psilocybin in the treatment of MDD, this trial did not report a significant difference in the primary outcome among the two groups. Secondary outcomes with other depression rating scales favoured psilocybin to escitalopram, but the analyses were not corrected for multiple comparisons and no conclusions can be drawn from the results. More RCTs are needed to further evaluate the efficacy of psilocybin in MDD.

The safety profile of psilocybin in the treatment of MDD is becoming established. Multi-centre phase 2 trials for psilocybin in MDD are currently in progress (NCT03775200, NCT03866174, NCT04670081, NCT03429075) and will likely give a more robust indication of efficacy. COMPASS Pathways recently announced results from a multi-centre phase IIb trial of psilocybin therapy in 233 participants with treatment-resistant depression (COMPASS Pathways, 2021). These results are not yet independently verified or peer-reviewed. A single dose of psilocybin 25 mg was reported to result in a rapid and enduring reduction in depressive symptom severity after 3 weeks, and nearly 25% of the patients had sustained response at 12 weeks. The majority of treatment-emergent adverse events occurred and resolved on the day of psilocybin administration. Suicidal ideation and behaviours were reported, all occurring in participants who were non-responders to psilocybin. Taking into account the timing and circumstances of adverse events including suicidal behaviours, psilocybin was reported to be generally well-tolerated in this trial (COMPASS Pathways, 2021). In comparison with brexanolone in PPD, psilocybin therapy appears to have similar rapid reduction in depression scores, with enduring response at 6 months. Phase 3 trials of psilocybin will give a better indication of psilocybin efficacy in treatment of depression.

## Psychedelics postpartum

There are no studies to our knowledge, animal or human, examining the safety of psychedelics postpartum, particularly in breastfeeding. In general, safety data for recreational drugs in breastfeeding is limited, with evidence largely coming from case reports (Anderson, 2018). The concentration of any drug in breast milk is influenced by factors such as maternal plasma concentration, maternal plasma protein binding, size of the drug molecule, degree of ionisation, and lipid solubility (Hotham and Hotham, 2015).

Breast milk drug concentration is found to be concordant with maternal plasma drug concentration (Hotham and Hotham, 2015). Peak plasma concentration of psilocin, the active metabolite of psilocybin, is reported to occur 105 ± 37 min after oral psilocybin administration (Hasler et al., 1997). The elimination half-life of psilocin is 3 h, indicating that, 48 h after oral administration of psilocybin, all but 0.0016% of psilocin will be eliminated (Brown et al., 2017; Passie et al., 2002). One study found psilocin to be undetectable in urine 24 h after oral administration (Hasler et al., 2002). Plasma protein binding also determines drug transfer into breast milk, with free unbound drugs diffusing readily (Hotham and Hotham, 2015). Psilocybin and psilocin both bind to human serum albumin, which suggests they are less likely to diffuse into breast milk (Khastar et al., 2020). Most drugs cross into breast milk in an unionised form, and milk, being slightly more acidic than plasma, attracts weak bases (Begg et al., 2002). Psilocin, with a pH of 5.2, is acidic and less likely to pass into breast milk (National Centre for Biotechnology Information, 2021).

Molecular size and lipid solubility also determine drug diffusion into breast milk. Low molecular weight drugs, such as psilocin, can cross readily into breast milk (Hotham and Hotham, 2015). Psilocin is more lipid-soluble than psilocybin, suggesting that it can diffuse readily into breast milk (Tylš et al., 2014). Psilocybin can transiently increase prolactin during peak effects, but levels return to baseline 5 h after oral administration, leaving no effect on breast milk production (Hasler et al., 2004).

Another factor determining the risk of infant adverse effects through drug exposure in breast milk is the age of the infant. About 78% of drug-related adverse effects occur in breastfeeding infants aged under 2 months, and only 4% of adverse effects are noted in infants older than 6 months (Anderson et al., 2003).

The pharmacokinetics of psilocybin and psilocin in breastmilk are yet to be examined in trials. However, current knowledge can be extrapolated to indicate that the lipophilicity of psilocin and its low molecular weight may allow for transfer into breast milk, while its acidity and binding to serum albumin suggest it is less likely to pass into breast milk. In the absence of pharmacokinetic evidence for psilocin in breast milk, maternal plasma concentration of psilocin may be the most useful indicator of breast milk concentration, with evidence to suggest that almost all psilocin will be eliminated by 48 h after administration. Including women beyond 6 months postpartum and advising abstention from breastfeeding for a 48-h period after psilocybin administration may reduce potential risks to the infant in future clinical trials.

## Therapeutic rationale for the use of psychedelics in the treatment of PPD

PPD has, at its core, a profound sense of maternal ‘disconnection’ – from the self, from the infant, and from the support network (Knudson-Martin and Silverstein, 2009). Themes that emerge from qualitative analyses of PPD symptoms include a deep sense of isolation, detachment from the infant, withdrawal from family and friends, an overwhelming sense of shame and guilt, and feelings of inadequacy as a mother (Beck, 2020; Holopainen and Hakulinen, 2019; Knudson-Martin and Silverstein, 2009). Reduced self-compassion, decreased maternal role gratification, increased self-criticism, maladaptive beliefs about motherhood, and negative perceptions of the infant are often associated with PPD (Felder et al., 2016; Sockol and Battle, 2015; Sockol et al., 2011). Bearing in mind these negative cognitions, PPD is associated with lower maternal sensitivity towards the infant (Bernard et al., 2018), which can impact on infant development, behaviour and mental health in later years (Bernier et al., 2019; Deans, 2020).

Psychedelics induce altered states of consciousness and can, in a dose-dependent manner, elicit mystical-type experiences, feelings of ‘oneness’ or interconnectedness, feelings of sacredness, transcendence of space and time, and a strong positive mood (Griffiths et al., 2006, 2018; James, 1902; MacLean et al., 2011). A core feature of the mystical-type experience is a sense of unity, an experience of becoming one with all that exists (Stace, 1960). Mystical-type experiences are associated with enduring improvements in depression and anxiety outcome measures in psilocybin trials and are thought to mediate these positive effects (Griffiths et al., 2016; Ross et al., 2016), although contrasting views also exist (Olson, 2020).

Along with this sense of unity and spirituality, a number of themes that emerge from qualitative studies of psychedelic experiences in participants with mental ill-health bear relevance to PPD. Acquiring insights into oneself, heightened self-awareness and a greater insight into one’s relationship with others are commonly cited by participants with anxiety and depression (Breeksema et al., 2020). Participants with treatment-resistant depression described a narrative of transformation from a pre-session ‘disconnection’ (from self, others and the world) to a renewed sense of ‘connectedness’ (to close family and friends, and all humanity) after psilocybin dosing (Belser et al., 2017; Watts et al., 2017). In the same cohort, participants reported gaining a fresh perspective on their lives, with increased self-compassion, an improved sense of self-worth, and a transition from emotional avoidance to acceptance (Watts et al., 2017). This sense of openness and acceptance was reported to persist for months in some participants (Watts et al., 2017). Increased self-compassion and self-acceptance have also been reported in psychedelic trials of participants with cancer and alcohol use disorder (Bogenschutz et al., 2018; Malone et al., 2018).

In addition to enhancing the experience of connectedness, LSD is associated with an increase in oxytocin levels in healthy participants, thought to be mediated through 5-HT2AR agonism (Holze et al., 2021). A systematic review examining the role of oxytocin in parent-infant attachments in infancy reported a positive correlation between maternal oxytocin levels and mother–infant interactions, and mothers with higher oxytocin levels were reported to have higher sensitivity in their infant interactions (Scatliffe et al., 2019). This hormonal effect of psychedelics, in conjunction with their psychological effects, may have a role in improving maternal sensitivity and the mother–infant relationship. Trials examining the relationship between psilocybin and oxytocin levels would be helpful in this regard.

An important aspect of psychedelic treatment is psychedelic-assisted therapy, both for ensuring safety and maximal benefit of the treatment for the patient. Psychedelic-assisted therapy involves preparation sessions with the therapist before the dosing session and support during the dosing session, followed by integration sessions after dosing. Preparation sessions are important for maximising the potential benefits of a psychedelic experience and integration is thought to prolong the positive effects of psychological transformation (Watts and Luoma, 2020). Contemporary models of psychedelic-assisted therapy are, in part, grounded in the principles of Acceptance and Commitment Therapy (ACT), a third-wave behavioural therapy emphasising acceptance of internal events in order to promote psychological flexibility and reduce experiential avoidance (Hayes et al., 2006). ACT has also shown promise in the treatment of perinatal mood and anxiety disorders (Bonacquisti et al., 2017; Waters et al., 2020).

**Figure 1.**
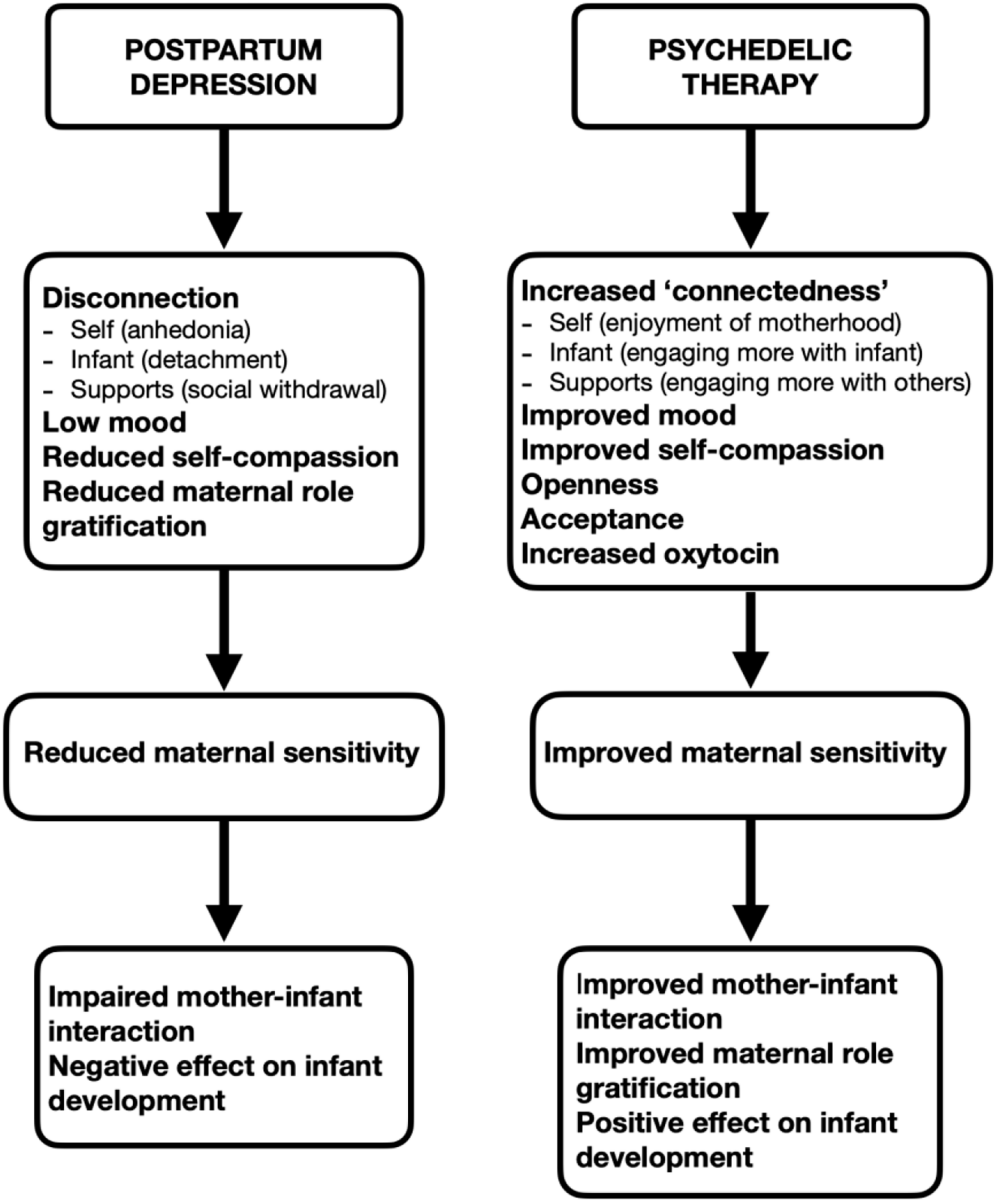
Therapeutic rationale for psilocybin therapy in the treatment of postpartum depression.

Taken together, psychedelics may, through mystical-type experiences, promote a sense of ‘reconnection’ for the mother in PPD. This increased sense of ‘connectedness’ can improve her connection with herself, her infant, and her support systems, which can have a positive effect on the mother–infant relationship [Figure 1]. Increase in oxytocin levels with psychedelic therapy may have a role in improving maternal sensitivity and mother–infant attachment. Furthermore, psychedelics, through increasing a sense of openness, acceptance and self-compassion, can foster an improvement in maternal self-compassion and role gratification, which can in turn enhance maternal sensitivity and the mother’s relationship with her infant (Fernandes et al., 2021).

## Discussion

There is increasing evidence for PPD being a distinct disorder to MDD in aetiology and neurobiology, although with some overlapping features. Poor response to treatment and treatment resistance is seen with conventional MDD treatment in PPD, highlighting its distinct therapeutic needs. Exploration of treatment with novel mechanisms is necessary to improve the mental health and wellbeing of women with PPD and their infants.

Psilocybin, with a favourable safety profile and propensity to promote openness, acceptance, and ‘connectedness’, in addition to positive effects on mood, may be a useful adjunct in the treatment of PPD. Maternal loss of connection, a core feature of PPD, can lead to a deep sense of isolation, lack of self-compassion and low mood, all of which can affect maternal sensitivity and infant development and mental health in later life. Psilocybin assisted therapy may have a role in addressing this ‘disconnection’ while improving maternal mood and maternal role gratification. Restoration of the mother’s connection with herself and her infant, along with an increased sense of acceptance, can help encourage self-compassion and address maladaptive beliefs about motherhood. Psychedelic-assisted therapy can act as a ‘container’ for this process of ‘reconnection’ to occur safely. It can also mitigate any adverse events in response to psilocybin and provide the necessary support and encouragement to take any lessons learned during the dosing session into the real world, practising them until they become habitual. The sense of acceptance and openness may, therefore, promote enduring positive change in the well-being of the mother–infant dyad.

The favourable safety profile of psilocybin, when administered in a therapeutic setting, is becoming evident in healthy participants and those with MDD (Carhart-Harris et al., 2016; Davis et al., 2021; Rucker et al., 2019). However, there is little research on the safety of psilocybin or other psychedelics in the perinatal period. Although the teratogenic potential of classic psychedelics appears low from observational studies (Cohen and Shiloh, 1977; Dishotsky et al., 1971; Long, 1972), in the absence of clinical trials of teratogenicity and in common with almost all other clinical drug trials, trials with classic psychedelics continue to exclude women who are pregnant, intending to become pregnant, or breastfeeding from participating.

There is no research into the safety of psilocybin in breastfeeding women, largely due to ethical concerns. However, in recent years, there has been a shift from systematic exclusion of breastfeeding women from clinical drug trials towards a more reasoned approach of inclusion while taking infant safety into consideration. The Council of International Organisations of Medical Sciences International Ethical Guideline for Health-related Research Involving Humans now promotes research designed to obtain knowledge relevant to the health needs of breastfeeding women, initiated after consideration of the best available data (Council for International Organisations of Medical Sciences, 2016). The latest International Council of Harmonisation Good Clinical Practice draft guideline gives consideration to the inclusion of breastfeeding women in clinical trials, highlighting that excretion of the investigational drug or its metabolites into breastmilk should be examined where applicable and feasible, with infants monitored for the effects of the drug (ICH Harmonised Guideline, 2019). Phase 2 trials continue to demonstrate the safety of psilocybin in the general population, and bearing in mind the potential benefit of psilocybin therapy on maternal wellbeing and the mother–infant relationship, there is a case for examining safety and feasibility of psilocybin in breastfeeding women. This would allow for broader inclusion of breastfeeding women in future psychedelic trials.

We propose that psilocybin may have a role in the treatment of PPD, particularly in engendering ‘reconnection’ of the mother to herself, her infant, and her support structures, and promoting positive enduring changes in the mother–infant relationship. We propose a pilot study to evaluate the safety and efficacy of psilocybin in the treatment of PPD. The increasing evidence base for safety of psilocybin in both healthy adults and those with MDD lays the foundation for pilot studies of psilocybin in the treatment of PPD, with appropriate precautionary and safety measures in place.

### Safety considerations

There are no studies, to our knowledge, of psilocybin use in breastfeeding. Excluding breastfeeding women from any future psilocybin trials in PPD would potentially disregard a large proportion of women with PPD, who are equally deserving of evidence-based treatments as any other group. As psilocybin is given as a single dose and found to be undetectable in urine by 24 h after administration, women may be advised to abstain from breastfeeding for a period of 48 h after psilocybin administration. By that stage, with an elimination half-life of 3 h, over 99.99% of psilocybin will have been eliminated from the maternal system (Brown et al., 2017). In addition, only 4% of adverse effects of breast milk drug exposure occur in infants older than 6 months (Anderson et al., 2003). Infant exposure and risk can be reduced by excluding breastfeeding women in the first 6 months postpartum, and advising women to abstain from breastfeeding for a 48-h period post-dose. These measures will reduce infant exposure to psilocybin and will allow breastfeeding women to participate in psilocybin trials.

## Limitations

A limitation of this review is that much of the evidence on psychedelics presented relates to the examination of psilocybin in the treatment of MDD. The evidence is derived from single-centre phase 2 trials with small sample sizes. Although these trials show signals of efficacy, larger multi-centre RCTs are awaited. There is no substantial data on the safety of psilocybin in the postpartum period, although safety of psilocybin has been demonstrated in healthy adults and those with MDD.

## Conclusion

Psychedelic-assisted therapy may be beneficial in treating one of the core features of PPD – a maternal loss of connection with herself and her infant – and can engender a sense of acceptance, which can promote enduring positive changes for the mother–infant dyad. It is reasonable, given the safety of psilocybin in adults and in MDD when used in a therapeutic setting, to examine safety of psilocybin in the postpartum period with a pilot study, which can then inform future studies of psilocybin in PPD.
